# A method to estimate the efficiency of gene expression from an integrated retroviral vector

**DOI:** 10.1186/1742-4690-3-51

**Published:** 2006-08-17

**Authors:** Hoi Ping Mok, Andrew Lever

**Affiliations:** 1 Department of Medicine, University of Cambridge, Level 5, Addenbrooke's Hospital, Hill's Road, Cambridge, CB2 2QQ, UK

## Abstract

**Background:**

Proviral gene expression is a critical step in the retroviral life cycle and an important determinant in the efficiency of retrovirus based gene therapy vectors. There is as yet no method described that can assess the efficiency of proviral gene expression while vigorously excluding the contribution from unstable species such as passively transferred plasmid and LTR circles. Here, we present a method that can achieve this.

**Results:**

Proviral gene expression was detected by the activity of the puromycin resistance gene encoded in the viral vector, and quantified by comparing the growth curve of the sample under puromycin selection to that of a series of calibration cultures. Reproducible estimates of the efficiency of proviral gene expression could be derived. We confirm that contamination from unstable species such as passively transferred plasmid used in viral vector production and unintegrated viral DNA can seriously confound estimates of the efficiency of transduction. This can be overcome using a PCR based on limiting dilution analysis.

**Conclusion:**

A simple, low cost method was developed that should be useful in studying the biology of retroviruses and for the development of expression systems for retrovirus based gene therapy.

## Background

Retroviruses include important human pathogens such as human immunodeficiency virus (HIV) and human T cell leukaemia virus 1 (HTLV-1). One of the features of the retroviral life cycle is integration, where the viral genome is incorporated into that of the host. An integrated viral genome is termed a provirus. A retrovirus can complete its life cycle only if gene expression from the provirus occurs. Studying the efficiency of proviral gene expression can potentially yield insights into the biology of this important class of viruses. In addition, retroviruses are increasingly used as vehicles for transgene delivery in gene therapy [1]. Recently, gene therapy associated insertional oncogenesis in a clinical trial [2] and in experimental models of fetal gene transfer [3] highlighted the importance of assessing the expression efficiency of the therapeutic vectors employed. Information on the efficiency of retroviral vector expression can aid in determining the number of integrations necessary to produce a therapeutic effect, thus improving the accuracy of the risk assessment [4,5], and limiting the dosage of vector used [6].

After infection, a retrovirus such as HIV reverse transcribes its RNA genome into double stranded cDNA. The cDNA has several possible fates. It can be integrated into the host genome, becoming a stable genetic element that can be passed onto daughter cells. Alternatively, it may circularise to a form bearing either one or two long terminal repeats (LTR). LTR circles are dead end products for the virus but soon after infection, they constitute the majority of the DNA species bearing the viral sequences [7]. LTR circles lack origins of replication and are diluted to extinction upon cell division. However, recent evidence suggest that they may be competent for gene expression [8-11]. Therefore in analysing the efficiency of proviral gene expression, methods must be devised which are able to distinguish between expression from a stably integrated provirus and from these unstable species.

The efficiency of proviral gene expression can be determined by quantifying the number of successfully transduced cells and the number of cells expressing the provirus. Viral titre is conventionally measured by examining the level of viral activity. Therefore a simple calculation of viral titre overlooks a population of cells that are successfully transduced but not expressing the virus. To address the proportion of successfully transduced cells, viral genetic elements have to be detected directly. Alu-PCR is a method that can detect the provirus but not other unstable viral DNA species [12]. It is based on the PCR amplification of an LTR and an Alu element in the host genome. However it is likely that the efficiency of Alu PCR varies with the distance between the provirus and the nearest Alu element in the host genome. Alternative methods may be needed to determine the proportion of successfully transduced cells.

The activities of non-selectable markers such as signals emitted by green fluorescent proteins (GFP) or luciferase are often used as proxies for measuring proviral gene expression. However, the levels of these signals do not necessarily correlate with proviral activity. We have previously demonstrated that the level of fluorescent signal arising from the incoming virus and from passively transduced GFP can be considerable [13]. In addition these signals can arise from the expression from unintegrated species. The latter can be excluded by using a selectable marker. To survive and proliferate in an antibiotic, a cell must express the appropriate antibiotic resistance gene, and pass the selectable marker onto daughter cells. Thus only gene expression from stably integrated provirus, but not unstable cDNA species, is included. However, the use of selectable marker is often limited by the on/off nature of the read out.

In this report, we present a simple method that can be used to estimate the efficiency of proviral gene expression *in vitro*. The proportion of cells that were successful infected and the fraction expressing a puromycin resistance gene encoded by the vector were separately determined. A method based on limiting dilution was used to determine the proportion of cells expressing the provirus. It required significant cell proliferation after dilution, thus ensuring that the signals detected arose from stable DNA species. Proviral gene expression was determined by assessing the proliferation of transduced cells in puromycin containing medium at a level toxic to untransduced cells.

## Results

An HIV-1 vector (HVP) with a LTR driven puromycin resistance reporter was utilised in this study. HVP is a plasmid that contains the proviral sequence based on the HIV-1 strain HXB2 (Genbank accession number: K03455). The provirus contains the packaging signal ψ, allowing the RNA produced to be packaged into the resultant vector in the producer cells. The provirus in HVP was inactivated by number of deletions: the viral gene *pol *was truncated, *nef *and part of *env *was deleted; *nef *being replaced by the puromycin resistance gene. In the producer cells, the viral vector was produced with two other helper plasmids supplying the polymerase gene products and the pantropic VSV-G envelope that pseudotypes the vector *in trans*. The vector produced was used to transduce Jurkat T cells, a human T cell line derived from a lymphoma [14]. The deletions in the vector genome ensured single round replication kinetics.

### Optimising proviral detection by PCR

**Figure 1:**
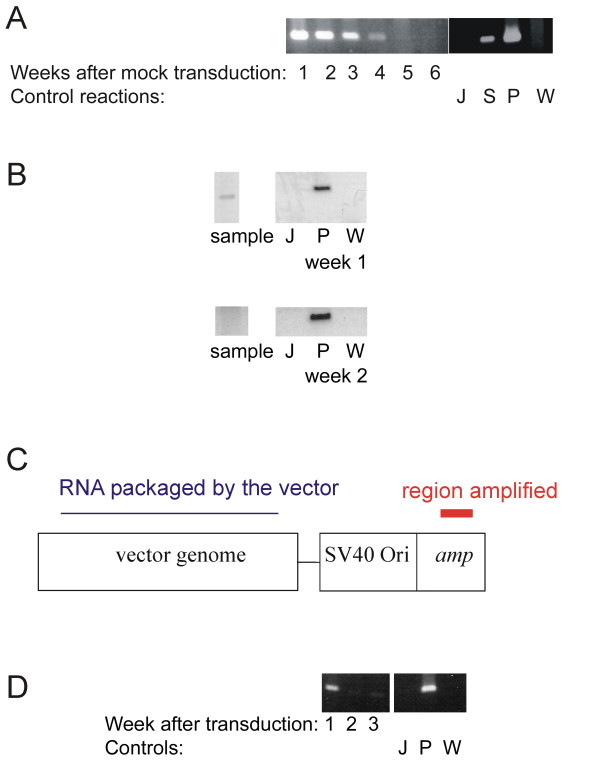
**Estimating the proportion of successfully transduced cells**. **a**. Significant transfer of plasmid occurred in the transfection – transduction process. Cos-1 cells were transfected with HVP without any helper plasmid using the calcium/BBS method. The supernatant was used to mock transduce Jurkat cells. LTR could be detected by PCR using primers NI2F and NI2R (see Methods) for at least four weeks after mock transduction. Control reactions were performed. DNA from untransduced Jurkat cells (J) and transduced, puromycin selected cells (S) was used as negative and positive extraction controls respectively. Reactions were also performed on HVP plasmid (P) or water (W) as positive and negative controls respectively. **b**. The method of transfection affects the extent to which passive transfer of plasmid occurs. The experiment was conducted as in (a), transfection was performed by the DEAE dextran method instead of the calcium/BBS method. LTR could be detected by PCR one week after mock transduction, but was no longer evident in the second week. Control reactions were performed, labelled as in (a). **c**. The ampicillin resistance gene is a target that can be used to detect passively transferred plasmid. A simplified, linearised diagram of the plasmid HVP is shown. The ampicillin resistance gene can be amplified by PCR (highlighted in red) using primers AmpF and Amp R (see Methods) to detect passively transferred plasmid, as it is distinct from the region coding for the viral vector, from which transcribed RNA could be packaged. **d**. Plasmid persists in transduced cells for at least three weeks. DNA was extracted from transduced cells. PCR for the ampicillin resistance gene was performed. A faint band can just be appreciated at three weeks post transduction.

Sensitive detection of the provirus was necessary to identify cells that were successfully transduced. However, PCR detection of the provirus was complicated by artefacts resulting from the unavoidable detection of passively transferred plasmid and unstable viral species such as LTR circles. Mock transduction experiments were conducted to evaluate the extent of this problem. Cos-1 cells were transfected with the vector genomic plasmid HVP without the helper plasmids. The supernatant was harvested and used to mock transduce Jurkat cells. When the producer cells were transfected by the calcium BBS method, passively transferred plasmid could be detected up to five weeks after mock transduction by PCR specific for the LTR (figure 1a). When the DEAE-dextran method was used for transfection, plasmid could be detected for the first week after transduction (figure 1b). In conventional transduction experiments, the vector is often prepared by concentrating the supernatant pooled from several dishes of transfected cells. To estimate the extent of plasmid transfer to target cells in this scenario, PCR was performed to detect the ampicillin resistant gene. Only proviral RNA transcribed from the genomic plasmid HVP will be specifically packaged into the vector. This is distinct from the region coding the ampicillin resistant gene (figure 1c). Plasmid could be detected for the first three weeks after transduction (figure 1d) but not by the seventh week (data not shown). Therefore passively transferred plasmid can lead to an overestimate of the proportion of cells harbouring provirus as detected by direct PCR, especially at early time points after transduction when using vectors produced by the calcium BBS transfection method.

**Figure 2:**
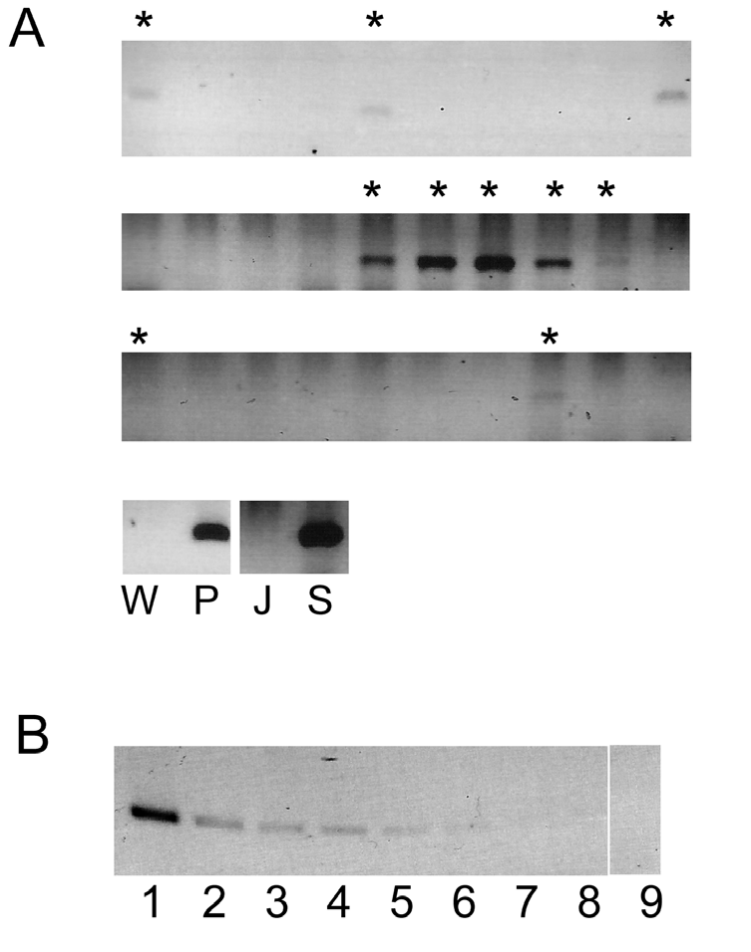
**a**. An example of the PCR read out after limiting dilution. Cells were seeded at an average of 125 cells per well on a 96 well plate (see Table 1 on the derivation of the number of cells per well). Thirty samples, each representing on average 125 cells, were expanded to 10 ml cultures. PCR was performed to detect the LTR using primers NI2F and NI2R (see Methods). Ten samples were positive (asterisked). To control for the quality of DNA extraction, puromycin selected cells (lane S) were included as a positive extraction and PCR control, while untransduced Jurkat cells were used as the corresponding negative control (J). Plasmid HVP (P) and water (W) were used as positive and negative reaction controls respectively. The proportion of cells containing a provirus is 10/(30 × 125) = 0.267%. A further refinement of this estimate is to take into account that each positive signal could arise from more than one cell containing the provirus and their distribution into each well is essentially random. In this case the average number of cells containing the provirus in each well would be -ln(20/30) = 0.4054. Since each well represented 125 cells, the proportion of successfully transduced cell was 0.4054/125 = 0.324%. **b**. The concentration of plasmid HVP was determined by spectrophotometry, which was then diluted and subjected to PCR. The amount of plasmid input from lanes 1 to 8 are: 10 pg, 1 pg, 100 fg, 50 fg, 33 fg, 25 fg, 13 fg, and 10 fg respectively. Lane 9 is a negative control, amplifying water only. The limit of detection was at lane 6 with the DNA input of 25 fg, corresponding to an input of approximately 2300 copies of plasmid.

To avoid the detection of passively transferred plasmid or unintegrated viral species, a limiting dilution based strategy was used. Cells were seeded in small numbers (0.1 to 100) into 96 well plates. They were then re-expanded into 10 ml cultures of more than a million cells per millilitre. DNA was extracted for PCR detection of the LTR. This method required a significant proliferation of cells before DNA isolation and PCR detection. Unintegrated DNA species would be diluted to extinction in the expansion process, allowing only integrated constructs to be detected. Figure 2a shows an example of the read out. The regions flanking the provirus in the plasmid HVP (a region that would not generate RNA that could be specifically packaged into the vector) could not be amplified from the samples positive for the LTR (data not shown), confirming that only stable species were detected. The proportion of cells that were successfully transduced was determined mathematically. In this transduction, only approximately 0.3% of the cells contained the provirus.

One potential source of error was the estimation of the number of cells seeded in each well, which might be different from the target number due to counting and pipetting errors. Thus the actual number of cells plated per well could not be assumed to be the number of cells counted in the plating process. Table 1 shows the method by which the actual number could be derived mathematically using the Poisson equation.

**Table 1:** An example of the calculation used to estimate the proportion of cells that contained the provirus.

Dilution	(1)	(2)	(3)	(4)	(5)	(6)	(7)
Dilution from (1) (*d*)		2	10	20	100	200	1000
Number of wells in which cell growth occurred	30	30	18	18	19	13	3
Number of wells in which cell growth did not occur	0	0	0	0	5	17	27
Proportion of wells that did not result in cell growth (*f*(*x*))	0/30	0/30	0/18	0/18	5/24 = 0.208	17/30 = 0.566	27/30 = 0.9
Mean number of cells (*m *= -ln *f*(*x*))					1.568	0.567	0.105
Estimated average number of cells per well in dilution (1) (*m *× *d*)					156.8	113.4	105

Cells from dilution (1) were amplified into 10 ml cultures and DNA was extracted for PCR detection of provirus, and the results are shown in figure 2. At higher dilutions (dilutions 5, 6 and 7), a proportion of wells showed no cell growth, allowing the average number of cells seeded in each well to be determined by the Poisson formula. This was used to estimate the average number of cells represented by each sample in dilution (1). In this case, the average number of cells represented is 125 (the average of 156.8, 113.4 and 105).

Another potential source of error was the sensitivity of the PCR. To employ this method the reaction has to be sufficiently sensitive to detect one provirus in the number of cells seeded in the sample. A theoretical limit of detection was obtained by dilution of plasmids to the limit of detection (figure 2b). One could expect that, if the limiting factor of detection is the sensitivity of the PCR, the frequency of positive signals would not decline as the number of seeded cells decreased, as each cell harbouring a provirus would now represent a larger proportion of the input DNA mix. This was not the case (data not shown). Clonal analysis was also performed. In this cell clones were derived from transduced cells, cultured, and pooled into samples containing no more than 15 clones. DNA was then extracted and PCR performed. If the sensitivity of PCR was a major limiting factor for the detection of a positive signal, one could predict that more samples would now be positive, as positive samples now constitute a larger proportion of the DNA input. Instead only one out of 152 clones screened contained the provirus. Together these observations suggest that the low frequency of detection in figure 2a was not due to poor sensitivity of the PCR reaction.

### Determining the proportion of cells that displayed antibiotic resistance

**Figure 3:**
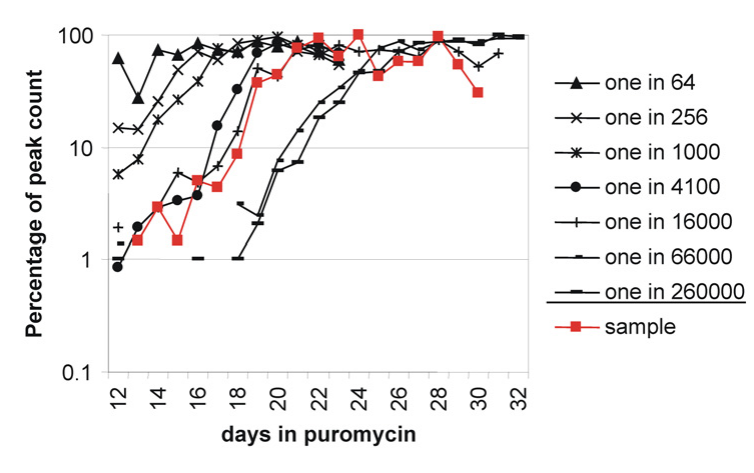
Estimating the proportion of cells that were puromycin resistant. A series of cultures, each containing 2.1 × 10^6 ^cells, with different proportions of puromycin selected cells and untransduced Jurkat cells (as indicated in the legend) were challenged with puromycin. Cell counts were monitored over time. An orderly change can be appreciated, with cultures containing higher proportions of puromycin resistant cells giving higher cell counts at an earlier time point. An equal number of cells from the sample were challenged with puromycin (curve in red). From this it can be estimated that about one in 16000 cells of this sample was puromycin resistant.

**Figure 4:**
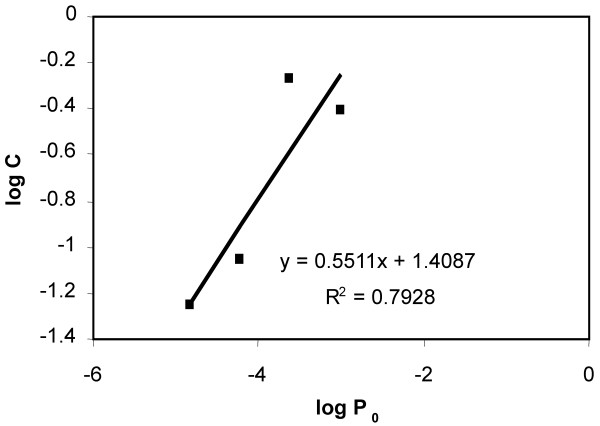
An example of the calculation to estimate the proportion of cells resistant to puromycin. Data from table 2 was plotted into a graph. In the graph, the vertical axis (*y*) is log*C*, the horizontal axis (*x*) is log*P*_0 _(see table 2). From the linear equation derived from the graph, . Therefore, after 21 days of selection in puromycin, the proportion of puromycin resistant cells in the original sample *P*_0 _and the cell count *C *is related by the equation . This relationship allowed the proportion of puromycin resistant cells in the sample to be determined. A more accurate estimate was obtained by averaging the *P*_0 _of the sample obtained by similar calculations performed for cell counts obtained on different days of culture in puromycin.

When cultured in the presence of puromycin, only cells harbouring an active provirus would be puromycin resistant and able to survive and proliferate. A family of cultures containing different proportions of puromycin selected cells and transduced cells was set up. A growth curve was obtained for each culture. This family of curves was superimposed onto the growth curve of the sample, allowing the proportion of puromycin resistant cells to be estimated. As shown in figure 3, an orderly relationship was observed; with cultures containing larger proportions of seeded puromycin resistant cells proliferating detectably earlier under selection. The proportion of cells that were puromycin resistant in the sample could be determined by superimposing its growth curve onto those of the calibrating cultures. Direct comparison of the growth curves gave an estimate bounded by the two calibration curves adjacent to the growth curve of the sample. A more precise estimate was obtained mathematically (table 2 and figure 4) by plotting a function of cell count against a function of the proportion of puromycin resistant cells seeded using data from the calibration cultures for each day of puromycin selection. An equation relating the cell count to the proportion of puromycin resistant cells seeded could be obtained for each day. The proportion of cells resistant to puromycin in the sample was determined from the equation. This was performed for each day on which data were available for the sample, and the proportion of puromycin resistant cells was an average of this series of estimates.

**Table 2:** An example of the data used to estimate the proportion of cells that were resistant to puromycin.

Proportion of puromycin resistant cells seeded (*P*_0_)	Log(*P*_0_)	Proportion of peak count (*C*_*T*_)	Log(*C*_*T*_)
1/1024	-3.010	0.3896	-0.4093
1/4100	-3.612	0.5368	-0.2701
1/16400	-4.214	0.0871	-1.059
1/66000	-4.816	0.0559	-1.252

The table shows the data obtained 21 days after cells were cultured in the presence of puromycin. Cultures containing different proportions of puromycin resistant cells and untransduced Jurkat cells (*P*_0_) were mixed and cultured under puromycin selection. The cells were counted daily, and the cell count at day 21 was expressed as a proportion of the peak count reached by the culture (*C*_*T*_). The data was plotted onto a graph (Figure 4). The data in this table are not directly comparable to that shown in figure 3 because the total numbers of cells used in the calibration cultures were different.

Two sets of calibration experiments were conducted. Data from different sets of experiments were comparable (data not shown). When the proportion of puromycin selected cells seeded was very low such that in theory only a small number (2–5) of cells in the culture were puromycin resistant, the probability of proliferation was essentially stochastic (data not shown). Although there was some variation in the absolute proportion of cells resistant to puromycin when different preparation of viral vectors were used in transduction, these proportions reflected the dilution of the vector preparation used in two independent experiments (table 3). Together these observations assured that the method employed was quantitative.

**Table 3:** Proportion of cells that were puromycin resistant after transduction reflected the dilution of the vector.

Experiment	Time after transduction (week)	Proportion of cells resistant to puromycin		Ratio of neat: ten fold dilution
			
		neat	1/10 dilution	
1	1	0.189	0.0131	14.4
	5	0.0111	7.73 × 10^-4^	14.9
2	2	l.64 × 10^-3^	2.64 × 10^-4^	6.21

Two independent experiments were conducted. Different vector preparations were used to transduce Jurkat T cells. In each experiment, transduction was conducted using both the neat vector stock and a 1/10 dilution of the preparation. The proportion of cells that were puromycin resistant was determined, as indicated. The absolute proportions of cells resistant to puromycin were different, reflecting the fact that different vector stocks were used. However, within each experiment, the proportion of cells resistant to puromycin was 6.21 to 14.9 fold higher when the neat stock was used, broadly reflecting the 10 fold dilution of the vector preparation used in transduction.

Five independent experiments were performed to determine the proportion of active proviruses after successful transduction in this transduction system. The values obtained ranged from 2.9 to 23%. In four of the five experiments, however, the range was 2.9 to 5.9% (manuscript in preparation). Therefore it was possible to obtain reproducible estimates on the efficiency of proviral gene expression using this method.

## Discussion

In this report, we present a method to estimate the efficiency of proviral gene expression of retroviral vectors. Particular attention was focused on excluding unstable DNA species such as LTR circles or the plasmids employed to produce the viral vector from analysis. A limiting dilution based method requiring significant proliferation of cells was devised, allowing unstable species to be diluted to extinction before proviral detection by PCR. Gene expression was detected by assessing the population growth when cells were cultured under puromycin. Since the puromycin resistance gene must be passed onto the daughter cells for them to survive and proliferate, this method ensured that only gene expression from stably integrated species was included in the estimate. This method yielded reproducible estimates on the efficiency of proviral gene expression from an HIV-1 vector in four out of five experiments. As with all other methods of gene expression determination, puromycin resistance measures a threshold expression instead of bona fide transcription. However other methodologies such as fluorescence analysis (threshold set at the point where the fluorescence signal departs from autofluorescence) or RNA measurement (threshold at limit of detection of RNA) do not allow for the selection of stable, non-transient gene expression.

This method is particularly suitable in systems where the efficiency of transduction or proviral gene expression is low. The proportion of cells expressing the provirus was assessed by examining the growth curve of the sample under selection, and comparing that with a family of cultures each with different proportion of drug selected cells. A number of factors could affect the results, such as spontaneous cell death, evaporation of media, limitation of nutrient and the accumulation of metabolic waste products in the culture. However, these factors would similarly affect both the calibration cultures and the sample, allowing a valid, direct comparison of the growth curves.

To obtain a more precise estimate, an idealised mathematical formula was used. An idealised formula relating the initial proportion of cells resistant to puromycin and the cell count observed was derived on different days after selection. While this method allows for a more precise estimate, it is also more susceptible to the confounding elements mentioned above. Perhaps one manifestation of the influence of these confounding elements was the fact the predicted gradient of the formula should be 1, while the actual gradient varied. By empirically re-deriving this formula on multiple days after selection rather than applying one idealised formula across multiple data sets, both errors arising from the underlying assumptions and random errors that could arise from relying on a single measurement were minimised.

There are several drawbacks to this method. It is labour intensive, slow, and relatively imprecise. It relies on the use of a drug resistance marker on the viral vector, limiting the scope of its application. The PCR here is optimised for the detection of the HIV-1 LTR. In practice, the reaction has to be designed for each application and the sensitivity of the reaction used determined. One important assumption made was that the frequency of infection of target cells is random, thus permitting statistical analysis. The advantages are that the method is simple and inexpensive. It ensures that only true proviral gene expression is measured. It should be useful as a research tool in studying proviral gene expression of retroviruses, and as a method to evaluate vectors and in designing promoter-enhancer systems for retroviral gene therapy applications.

## Conclusion

A method was developed to estimate the efficiency of proviral gene expression. This low cost method rigorously excludes confounding elements that can arise from other steps of the retroviral life cycle. It should be useful in the study of retroviral transcription and expression of gene therapy vectors.

## Materials and methods

### Cells and puromycin selection

Cos-1 cells [15] were maintained in Dulbecco's modified essential medium (Gibco) containing 10% fetal calf serum (FCS) (Gibco) and 5% penicillin-streptomycin (Gibco). Jurkat cells [14] were maintained in RPMI1640 medium (Gibco) also containing 10% FCS and 5% penicillin-streptomycin. Where puromycin selection was applied a concentration of 0.5 μg/ml was used.

### Transfection

To transfect cells by the calcium BBS method, Cos-1 cells were plated onto 10 cm dishes. The media was aspirated and replaced. Two to four hours later, the DNA mix was prepared. 737 μl of 2×BBS (50 mM N, N-bis(2-hydroxyethyl)-2-aminoethanesulphonic acid (BES), 280 mM NaCl, 1.5 mM Na_2_HPO_4_, pH 6.94–6.96), 737 μl of water, an appropriate amount of the plasmid, and 72.5 μl of CaCl_2 _was mixed together. The mix was swirled, filter sterilised and allowed to stand for 20 minutes at room temperature for the DNA to precipitate. The mix was added dropwise to cells. The cells were incubated overnight at 37°C with in 3% carbon dioxide atmosphere. The media was replaced the next day, and the dishes were transferred to a 37°C incubator with a 5% carbon dioxide atmosphere.

To transfect cells by the DEAE dextran method, cells were plated onto 10 cm dishes. The dishes were rinsed twice with PBS. The DNA mix was prepared. The plasmid was added to 1.9 ml of PBS followed by 100 μl of stock diethylaminoethyl (DEAE) dextran (stock DEAE dextran: 10 mg/ml in 1 M Tris-HCl, pH 7.3–7.5, Amersham). The mix was filter sterilised. DNA mix was added to the cells. The cells were incubated at 37°C for 30 minutes. 5 ml of freshly prepared 80 μM chloroquine in serum free media was added. The cells were incubated at 37°C for a further 2.5 hours. The media was then aspirated, and the cells were shocked with 2 ml of 10% dimethylsulfoxide (DMSO) in serum free medium for two minutes. The medium with DMSO was aspirated and the cells were washed twice with serum free media, before being cultured in serum containing media and returned to the incubator at 37°C.

### Vector and transduction

HIV-1 vector was prepared by the simultaneous transfection of three plasmids pHVP [16], pΔp1 (a Gag/Pol expressing plasmid with a deletion in the major packaging signal [17] and pVSV-G into Cos-1 cells. The tissue culture supernatant of transfected Cos-1 cells was harvested 48 hours after transfection. Cell debris was removed by passing the supernatant through a 0.45 μm filter. In mock transduction experiments, the filtered supernatant was layered directly onto 10^6 ^Jurkat cells. In other experiments, the filtered tissue culture supernatant from up to eight dishes of transfected Cos-1 cells was used. 0.5 volumes of 30% polyethylene glycol 8000 (Sigma) (PEG, 30% PEG in 0.4 M NaCl) were added to the supernatant. The supernatant was mixed and left to precipitate overnight at 4°C. The PEG precipitated vector was centrifuged at 2000 revolution per minute (rpm) in a Falcon 6/300 centrifuge (rotor model 43124-129, MSE) for 40 minutes at 4°C. The pellet was resuspended in 0.5 ml of TNE (10 mM Tris-Cl, 150 mM NaCl, 1 mM EDTA pH7.5) and layered onto 0.5 ml of 20% sucrose in TNE. The mix was then ultracentrifuged at 40000 rpm in a Beckman TLA 55 rotor for 2 hours at 4°C. The resultant pellet was resuspended in media. To transduce Jurkat cells, the vector preparation was applied to 10^6 ^Jurkat cells, and the final volume of transduction was restricted to less than 2 ml. Cells were incubated in this small volume with the vector preparation for 24 hours.

### Limiting dilution and cell growth monitoring

Cells were counted at least four times, and mixed with measured amounts of media to generate stocks of 5 × 10^4 ^cells per millilitre. This stock was then further diluted to the concentration for the lowest dilution in the series (eg 500 cells/ml for 100 cells per well on a 96 well plate). Higher dilutions were obtained by further serial dilutions. 200 μl of the diluted culture was added to each well of 96 well plates. The tissue culture plate was examined regularly. Monitoring was stopped when no new cell growth could be observed after two consecutive weeks and the proportion of wells that eventually resulted in cell growth was noted.

To monitor Jurkat cell growth under selection, 10 μl of culture was mixed with 10 μl of trypan blue (Sigma) to exclude dead cells. The mix was added to a haematocytometer (Knittel Glaser, or Kova Glasstic, Hycor) and cells were counted under a microscope. The cell count was converted to cells per millilitre of culture.

### DNA extraction and PCR

Genomic DNA was also extracted from cells using a commercially available kit (DNeasy, Qiagen) following the protocol of the manufacturer.

Each PCR mix contained 500 nM of forward primer, 500 nM of reverse primer, 1× PCR buffer, 2 mM MgCl_2_, 2.5 units of Taq DNA polymerase (Sigma) and an appropriate amount of DNA template. In PCR for LTR, 5% DMSO (Sigma) was used as an enhancer. The reaction was made up to 50 μl with water. The primers used to detect HIV-1 LTR are NI2F (5'-cacacacaaggctgacttccct-3') and NI2R (5'-gccactccccagtccgccc-3'). The primers used to detect the ampicillin resistant gene are AmpF (5'-gataacactgcggccaactt-3') and AmpR (5'-ttgccgggaagctagagtaa-3'). The reactions were cycled either in a Perkin Elmer thermocycler (DNA Thermal Cycler, N801 0150) or a Techne thermocycler (Touchgene FTG05TD). Initial denaturation was conducted at 94°C for four minutes. Forty cycles of reaction were performed, each consisting of denaturation at 94°C for two minutes, reannealing at 58°C for 30 seconds and elongation at 72°C for one minute and 30 seconds. Final elongation was conducted at 72°C for seven minutes.

### Calculations

#### 1. Estimating the proportion of cells that contained the provirus

The proportion of cells that were successfully transduced could be estimated from the PCR data. If the number of positive samples were *a*, the total number of PCR samples is *n *and each sample represent *m *number of cells, the proportion of cells containing the provirus would thus be . However, considerable error could be introduced by limiting dilution. Thus, *m *has to be corrected by data obtained in limiting dilution. It was assumed that at high dilutions, the distribution of number of cells seeded in each well followed a Poisson distribution:



where *x *is the number of cells seeded in each well,

*m *is the mean number of cells seeded in each well,

*f*(*x*) is the probability of having *x *number of cells seeded in each well. When no cell growth could be observed, *x *= 0. *f*(*x*), which is now *f*(0), is the proportion of cells that did not result in cell growth. Therefore *m *of each dilution could be obtained:



*f*(0) = ***e***^-*m*^

ln *f*(0) = ln(***e***^-*m*^)

*m *= -ln *f*(0)

Samples from lower dilutions (higher number of cells per well) were often analyzed by PCR. The mean number of cells in each well was calculated from data of cell growth obtained from series of higher dilutions and corrected with the dilution factor.

A further correction was made in some estimates, when the proportion of samples with a positive signal was large, such that a significant number of them would represent two or more successfully transduced cells. The distribution of successfully transduced cells in each well of the 96 well plates from which the sample was derived was random. Thus, by Poisson distribution, the number of positive cells represented by each sample



Since the number of cells represented by each sample was the corrected *m*, the proportion of successfully transduced cells was thus



#### 2. Estimating the proportion of populations that were resistant to puromycin

Theoretically, after *T *days of culture in the presence of puromycin, the number of cells in the culture *N*_*T*_, the initial number of puromycin resistant cells *N*_0 _and the doubling time *t*_2 _were related by the equation



If the volume of the culture was constant then cell count *C *would be related to the total number of cells *N *by a conversion factor *a*. Thus:



The cultures were heterogeneous containing both puromycin resistant and sensitive cells. The total number of cells in each culture was held constant, rendering the fraction of cells that were resistant to puromycin *P *to be directly proportional to the number of cells that were puromycin resistant. Let *b *be the conversion factor:



A series of cultures with different *P*_0 _was obtained and population growth was monitored by daily counts. It would have been possible to plot *C*_*T *_and *P*_0 _directly. However, the values of these parameters were small and plotting logarithmic values does not alter the linearity of the relationship. On any fixed day *T *after culture, *T*, *b*, *a*, and *t*_2 _are all constants. Therefore  is a constant. The cell count for each calibration culture on each day was divided by the peak count reached. Data was removed if the values of *C*_*T *_were larger than 0.8 or smaller than 0.05, as they represent points that were affected by overcrowding or stochastic behaviour respectively. Graphs of log *C *against log *P*_0 _were plotted, from which the proportion of cells resistant to puromycin was estimated by the cell count, *C *of the sample. The proportion *P*_0 _was calculated on each day where usable data of the sample culture were available and then averaged.

## Competing interests

The author(s) declare that they have no competing interests.

## Authors' contributions

HPM and AML jointly conceived of the experiments and wrote the manuscript. HPM performed the experiments with advice from AML. Both authors read and approved the final manuscript.
